# Endogenous tetrahydrobiopterin in humans: circadian rhythm, sex, race, age, and disease status

**DOI:** 10.3389/fphar.2025.1701617

**Published:** 2025-12-05

**Authors:** Lan Gao, Neil Smith, Ronald Kong

**Affiliations:** PTC Therapeutics, Inc., Warren, NJ, United States

**Keywords:** 5,6,7,8-tetrahydrobiopterin (BH_4_), circadian rhythm, sex, race, age, phenylketonuria (PKU), primary tetrahydrobiopterin deficiency (PBD)

## Abstract

**Introduction:**

*6R*-L-erythro-5,6,7,8-tetrahydrobiopterin (BH_4_) is an essential cofactor for multiple enzymes, including phenylalanine hydroxylase (PAH). Exogenous BH_4_, or its natural precursor sepiapterin, is utilized to treat patients with phenylketonuria (PKU), a disease caused by PAH deficiency. This study aims to investigate correlation of endogenous BH_4_ concentrations with related factors, circadian rhythm, sex, race, age, and disease status.

**Methods:**

Predose or placebo treatment blood samples were collected in eight sepiapterin clinical trials from healthy adults and patients of all ages with PKU or primary tetrahydrobiopterin deficiency (PBD) to measure plasma BH_4_ concentrations. Graphic visualization, descriptive statistics, and analysis of variance were used to explore the relationship between participant characteristics and BH_4_ concentrations.

**Results:**

In total, 1175 BH_4_ measurements from 236 participants were analyzed, revealing a circadian rhythm of BH_4_ concentration. In healthy adults, BH_4_ had the lowest concentrations between 7:00 and 10:59 (geometric mean 2.06 ng/mL) and the highest between 19:00 and 22:59 (2.72 ng/mL). Asian participants exhibited the highest BH_4_ concentration (2.33 ng/mL), whereas comparable levels were observed in Whites and Blacks or African Americans (2.01 and 2.07 ng/mL, respectively). Endogenous BH_4_ in PBD patients was <0.5 ng/mL, while it was significantly higher in PKU patients (9.63 ng/mL for those >2 years). No age-dependent BH_4_ change was observed in healthy adults and participants with PKU >2 years. BH_4_ concentrations were higher in healthy adult males (2.18 ng/mL) than females (1.95 ng/mL), but not distinguishable between male and female patients with PKU.

**Conclusion:**

Circadian rhythm and significant differences between sexes and races in BH_4_ concentrations were observed in healthy adults. BH_4_ concentrations do not change with age in healthy adults and PKU patients >2 years. BH_4_ concentrations were relatively stable between 7:00 and 10:59, providing a window for measurements with minimal variation. The significant difference in BH_4_ concentrations between patients with PBD, patients with PKU, and healthy adults could be utilized as a diagnostic tool.

## Introduction

*6R*-L-erythro-5,6,7,8-tetrahydrobiopterin (BH_4_) is an essential cofactor and natural stabilizer for multiple aromatic amino acid hydroxylation enzymes, such as phenylalanine hydroxylase (PAH), tyrosine hydroxylase (TH), and tryptophan hydroxylase (TPH), alkylglycerol monooxygenase (AGMO), and nitric oxide synthase (NOS) (Thony et al., 2004; Thony et al., 2008; Komleva et al., 2025). Multiple inborn errors of metabolism are closely related to the biosynthesis and regeneration of BH_4_; therefore, it serves as an important drug target to treat metabolic disorders such as phenylketonuria (PKU), primary tetrahydrobiopterin deficiencies (PBD), and tyrosine hydroxylase deficiency (Werner-Felmayer et al., 2002; Werner et al., 2011; Werner, 2013; Fanet et al., 2021; Eichwald et al., 2023; Jung-Kc et al., 2024). Exogenously supplemented BH_4_ (sapropterin dihydrochloride) has been approved for the treatment of hyperphenylalaninemia (HPA) due to BH_4_-responsive PKU (Blau et al., 2010; Blau, 2013). Results from recent Phase 3 studies indicated that a broader population of patients with PKU could benefit from treatment with sepiapterin, a natural precursor of BH_4_ that was recently approved in the EU and US (Muntau et al., 2024; van Spronsen et al., 2025).

Despite the importance of BH_4_ in the pathology of BH_4_-dependent enzyme metabolic diseases, studies of natural fluctuation of endogenous BH_4_ in human were rarely reported. This could be due to the lability of BH_4_, which is subject to autoxidation under physiologic conditions (Mortensen and Lykkesfeldt, 2013; Telegina et al., 2018). BH_4_ represents 65%–80% of total biopterins in plasma, and large variations in biopterin concentration from 6.9 nmol/L to 23.6 nmol/L have been observed, which have been attributed to BH_4_ lability (Fukushima and Nixon, 1980; Fiege et al., 2004; Fekkes and Voskuilen-Kooijman, 2007). The lability of BH_4_ has been well recognized for >50 years, and it remains an active area of research today (Blair and Pearson, 1973; Buglak et al., 2021; Boulghobra et al., 2023).

Determining the plasma concentration of BH_4_ is not feasible without stabilization. Various antioxidants such as dithioerythritol (DTE), dithiothreitol (DTT), and ascorbic acid (Fiege et al., 2004; Fekkes and Voskuilen-Kooijman, 2007; Kaushik et al., 2024); acids to control pH, like meta-phosphoric acid, perchloric acid, and trichloroacetic acid (Mortensen and Lykkesfeldt, 2013); and chelating agents, such as ethylenediaminetetraacetic acid and diethylenetriaminepentaacetic acid, have been explored to stabilize BH_4_ in the biomatrix (Guibal et al., 2017). Depending on the procedures followed and the concentrations of reagents used, very different results were observed. However, it is generally agreed that lower pH reduces the rate of autoxidation, and the half-life of BH_4_ ranges from 15 min to 127 min, depending on the buffer and pH (Mortensen and Lykkesfeldt, 2013; Boulghobra et al., 2023). Accordingly, it is critical to stabilize BH_4_ in blood immediately after sample collection.

Additionally, the most widely used method to measure BH_4_ is the indirect method introduced by Fukushima and Nixon about half a century ago (Fukushima and Nixon, 1980). The method utilizes chemical oxidation under acidic conditions to convert both BH_4_ and 7,8-dihydrobiopterin (BH_2_) to biopterin, and oxidation under alkaline conditions to convert BH_2_ to biopterin and BH_4_ into pterin. Biopterin is quantified using high-performance liquid chromatography (HPLC) with fluorescence detection (FD). The difference in the amount of biopterin between the two HPLC-FD runs reflects the amount of BH_4_. The indirect method is not only tedious, but also subject to variations in oxidation conversion efficiency, which has been reported to be far less than complete, approximately 47% in human plasma (Zhao et al., 2009). Additionally, there is potential interference from the matrix and variability in multiple HPLC runs. The lower limit of quantitation (LLOQ) of the indirect method for plasma BH_4_ measurements is rarely reported, and it is generally assumed to lack adequate sensitivity for reliably measuring endogenous plasma BH_4_ concentration. Various adjustments have been made to improve the indirect method since its introduction, such as the direct measurement of BH_4_ with post-column coulometric oxidation by HPLC-FD (Hyland, 1985; Guibal et al., 2014) and HPLC mass spectrometry (HPLC-MS) (Fismen et al., 2012; Kim et al., 2012).

Recently, a highly selective and sensitive HPLC-MS method to quantify BH_4_ in human plasma has been developed and fully validated following Good Laboratory Practice guidance (Kaushik et al., 2024). Blood samples were placed in wet ice immediately after collection and stabilized within 10 min of collection with 10% ascorbic acid at a 100:11 (v:v) ratio to reach a final ascorbic acid concentration of 1%. Treated blood was centrifuged at 4 °C for 10 min at a centrifugal force of 2000-g within 30 min of blood sample collection. It was then stored at −80 °C and analyzed within the established stability period. During the process of blood sample collection, processing, and plasma sample analysis, samples were protected from light. The method has an LLOQ of 0.5 ng/mL for measuring BH_4_ concentrations in human plasma. The sensitivity and robustness of this HPLC-MS method were demonstrated during method validation, as well as by its application in multiple clinical studies, in which the accuracy and precision of every analytical run and incurred sample reanalysis were examined. The high specificity and sensitivity of this method made it feasible to investigate endogenous BH_4_ concentrations to detect small fluctuations in healthy volunteers and in patients with PKU or PBD.

In this study, data were pooled together from measurements of blood samples collected at predose or placebo treatment in healthy adults and patients of all ages with PKU or PBD from eight sepiapterin clinical studies. The aim of this study was to assess the impacts of circadian rhythm, sex, age, race, and disease status on endogenous BH_4_ concentrations to gain a better understanding of the molecule and its role in pharmacology.

## Materials and methods

### Data source and collection

Blood samples were collected from healthy volunteers, patients with PKU, and patients with PBD (due to 6-pyruvoyl-tetrahydrobiopterin synthase [6-PTPS] deficiency) from eight clinical studies and plasma BH_4_ concentrations were measured using a validated HPLC-MS method (Kaushik et al., 2024). The clinical studies included in this analysis are summarized in Supplementary Table S1. All clinical studies were conducted following Good Clinical Practice guidelines and in compliance with the principles of the Declaration of Helsinki. The protocols and informed consent forms were approved by local independent ethics committees or institutional review boards, and regulatory agencies, as required. Signed consent forms were provided by all participants prior to enrollment.

To reflect the endogenous BH_4_ concentrations, only data from samples collected prior to the first treatment or predose samples of each treatment period following a minimum washout of 4 days if there were multiple treatment periods, and samples following placebo treatments were included in the analysis.

### Data analysis

Data were binned by hour following the 24-h clock period according to the actual sampling time. For example, data from samples collected from 7:00 to 7:59 would be binned for the 7-h period. If data from multiple samples collected from the same participant in the same hour period were available, the geometric mean would be calculated and used for the analysis. Hence, a subject had only one value in any specific hour period.

Following the hourly binned analysis, data were further binned by 4-h time blocks, including 7:00–10:00 (7:00 to 10:59), 11:00–14:00, 15:00–18:00, 19:00–22:00, except for the 23:00–06:00 time block. Since there were only two data points available in the 23:00–06:00 time block, it was not further divided. If multiple values were available from the same participant in the same time block, the geometric mean would be calculated and used for the analysis. Hence, a subject had only one value in any 4-h time block.

Descriptive statistics were used to summarize data by categories. Analysis of variance (ANOVA) was used to compare the differences between categories.

Sex, age, race, and disease status comparisons were conducted based on data collected during the 7-11 time block, as this time block contained the richest data; later analysis also suggested that endogenous BH_4_ concentrations were relatively stable during this period.

### Software

This analysis was conducted using R (version 4.3.3; R Foundation for Statistical Computing, Vienna, Austria) with R Studio (version 2023.09.1, Posit Software, PBC, Boston, MA, United States of America).

## Results

### Demographic characteristics and data summary

Data from eight clinical studies in adult healthy volunteers and participants of all ages with PKU or PBD were included in this analysis (Supplementary Table S1). Designs and results from these clinical studies have been previously reported (Smith et al., 2019; Gao et al., 2024a; Gao et al., 2024b; Gao et al., 2024c; Muntau et al., 2024; van Spronsen et al., 2025).

A total of 236 participants provided 1175 BH_4_ measurements for this study (Table 1). Among them, 167 participants were healthy volunteers, with a median (minimum, maximum) age of 32.0 (18.0, 56.6) years; seven participants with PBD (all due to 6-PTPS deficiency), with a median of age 11.0 (6.00, 20.0) years; and 62 participants with PKU, with a median age of 16.0 (0.380, 61.0) years. Overall, approximately equal numbers of male (48.7%) and female participants (51.3%) were enrolled in the studies. In healthy volunteers, males and females represented 49.1% and 50.9% of the population, respectively; in participants with PKU, males and females represented 46.8% and 53.2% of the population, respectively; and in participants with PBD, males and females represented 57.1% and 42.9% of the population, respectively. The majority of participants were White (67.4%), followed by Asian (16.1%) and Black or African American (9.3%). Participants with unidentified race or identified with multiple races (parents of different races) were grouped as Other (5.9%).

**TABLE 1:** Characteristics of participants and BH_4_ measurements.

Characteristic	HV	PBD	PKU	Overall
Number of participants	167	7	62	236
Age group, n (%)				
≥18 years	167 (100)	3 (42.9)	28 (45.2)	198 (83.9)
≥12 and <18 years	0 (0)	0 (0)	9 (14.5)	9 (3.8)
≥6 and <12 years	0 (0)	4 (57.1)	9 (14.5)	13 (5.5)
≥2 and <6 years	0 (0)	0 (0)	9 (14.5)	9 (3.8)
<2 years	0 (0)	0 (0)	7 (11.3)	7 (3.0)
Age				
Mean (CV%)	33.4 (28.5)	13.0 (45.3)	19.6 (81.2)	29.2 (45.4)
Median (min, max)	32.0 (18.0, 56.6)	11.0 (6.0, 20.0)	16.0 (0.4, 61.0)	30.0 (0.4, 61.0)
Race, n (%)				
Native American or Alaska native	1 (0.6)	0 (0)	2 (3.2)	3 (1.3)
Asian	25 (15.0)	2 (28.6)	11 (17.7)	38 (16.1)
Black or African American	22 (13.2)	0 (0)	0 (0)	22 (9.3)
Other	10 (6.0)	0 (0)	4 (6.5)	14 (5.9)
White	109 (65.3)	5 (71.4)	45 (72.6)	159 (67.4)
Sex, n (%)				
Female	85 (50.9)	3 (42.9)	33 (53.2)	121 (51.3)
Male	82 (49.1)	4 (57.1)	29 (46.8)	115 (48.7)
Number of measurements	1058	7	110	1175
Age group, n (%)				
≥18 years	1058 (100)	3 (42.9)	56 (50.9)	1117 (95.1)
≥12 and <18 years	0 (0)	0 (0)	25 (22.7)	25 (2.1)
≥6 and <12 years	0 (0)	4 (57.1)	9 (8.2)	13 (1.1)
≥2 and <6 years	0 (0)	0 (0)	13 (11.8)	13 (1.1)
<2 years	0 (0)	0 (0)	7 (6.4)	7 (0.6)
Sex, n (%)				
Female	567 (53.6)	3 (42.9)	45 (40.9)	615 (52.3)
Male	491 (46.4)	4 (57.1)	65 (59.1)	560 (47.7)
BLQ, n (%)				
No	1052 (99.4)	0 (0)	105 (95.5)	1157 (98.5)
Yes	6 (0.6)	7 (100)	5 (4.5)	18 (1.5)

BH_4_, 6R-L-erythro-5,6,7,8-tetrahydrobiopterin; BLQ, below the limit of quantification; CV, coefficient of variation; HV, healthy volunteer; PBD, primary tetrahydrobiopterin deficiency; PKU, phenylketonuria.

All healthy participants (*n* = 167) were adults (≥18 years). In participants with PBD, there were three adult participants and four children aged ≥6 and <12 years. In participants with PKU, there were 28 adults (≥18 years), 9 subjects each from age groups ≥12 and <18 years, ≥6 and <12 years, ≥2 and <6 years, and 7 children of age <2 years (Table 1).

Predose BH_4_ measurements for participants with PBD were all reported below the limit of quantification (BLQ; Table 1). Thus, they were treated as zero during the analysis. It is believed that this reflects the true value in patients with PBD, as suggested by the coherence of the data. This is also expected because autosomal recessive 6-PTPS deficiency in patients with PBD essentially blocks *de novo* BH_4_ biosynthesis (Opladen et al., 2020).

Additionally, there were six BH_4_ measurements from healthy volunteers and five measurements from participants with PKU reported as BLQ. These represented 0.6% and 4.5% of the data from healthy volunteers and participants with PKU, respectively (Table 1); both were <5% of the total measurements of each population. These data were deemed as outliers and were excluded from the analysis. Since these BLQ data consisted of <5% of total measurements, exclusion of these data had a negligible impact on the reliability of the analysis (Xu et al., 2011).

### Endogenous BH_4_ concentrations in healthy adult volunteers

In total, 1058 BH_4_ measurements were obtained from 167 adult healthy volunteers (Table 1). After hourly binning, there were 651 BH_4_ observations from 167 healthy volunteers. Most BH_4_ data points were collected between 7:00 to 22:59. There was only one data point each at 23:00 and 01:00. The overall geometric mean of BH_4_ was 2.29 ng/mL with 2.5% and 97.5% percentile of distribution of 1.12 and 3.73 ng/mL, respectively.

#### Circadian rhythm

**FIGURE 1:**
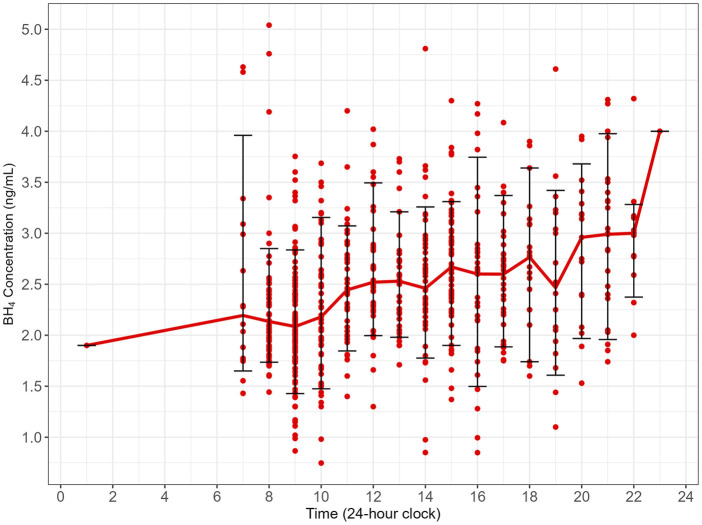
Variations in endogenous BH_4_ concentrations in healthy adult volunteers with time. Red dots: observed data; solid red line: median BH_4_ for each hour; error bar: 10% and 90% of observed data. BH_4_, *6R*-L-erythro-5,6,7,8-tetrahydrobiopterin.

A descriptive summary with the mean (standard deviation [SD]), geometric mean (geometric coefficient of variation [GCV%]), median with 2.5% and 97.5% percentile of distribution for each period were calculated and are summarized in Supplementary Table S2. The observed data and the trend of median BH_4_ concentration with time is illustrated in Figure 1.

There was a relatively wide distribution of endogenous BH_4_; the GCV% was approximately 30% across the hours. Nevertheless, median BH_4_ still illustrated a clear trend with circadian rhythm. BH_4_ concentration was the lowest in the morning and relatively stable between 07:00 and 10:59, then gradually increased during the day until the highest concentration was attained between 21:00 and 22:59 (Figure 1). There were only two data points available between 23:00 and 01:59, and there were no data available between 02:00 and 06:59 (Supplementary Table S2).

**FIGURE 2:**
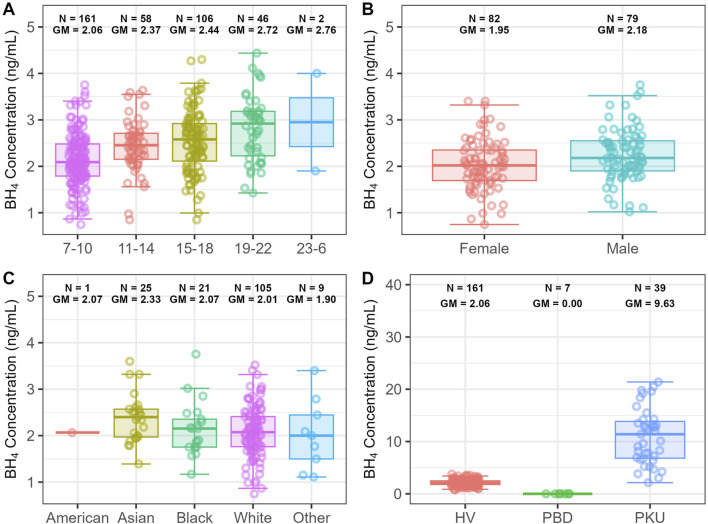
Box plots to compare endogenous BH_4_ concentration. **(A)** Between 4-h time blocks in healthy adult volunteers. **(B)** Between male and female healthy adult volunteers. **(C)** Between different races in healthy adult volunteers; **(D)** between populations aged >2 years with different disease statuses. BH_4_, *6R*-L-erythro-5,6,7,8-tetrahydrobiopterin; GM, geometric mean; HV, healthy volunteer; PBD, primary tetrahydrobiopterin deficiency; PKU, phenylketonuria; American, Native American or Alaska Native; Black, Black or African American.

An ANOVA was conducted after data were binned into the 4-h period time blocks, except for data in the 23:00–06:00 time block (23:00–06:59), which had only two BH_4_ measurements and was considered to have insufficient data for the comparison (Table 2). In the 7:00–10:00 time block, the geometric mean BH_4_ concentration was 2.06 ng/mL and increased to peak concentrations of 2.72 ng/mL in the night between 19:00 and 22:00. Compared with the 7:00–10:00 time block, BH_4_ was significantly higher for the rest of day in the 11:00–14:00 and 15:00–18:00 time blocks. BH_4_ further increased in the evening in the 19:00–22:00 time block, and the increase was significant compared with the 11:00–14:00 and 15:00–18:00 time blocks (Table 2; Figure 2A).

**TABLE 2:** Endogenous BH_4_ concentrations in healthy adult volunteers by 4-h time block.

Endogenous BH_4_ concentration, ng/mL	Time block (no. of measurements)					
	7:00–10:00 (*n* = 161)	11:00–14:00 (*n* = 58)	15:00–18:00 (*n* = 106)	19:00–22:00 (*n* = 46)	23:00–06:00^a^ (*n* = 2)	Overall (*N* = 373)
Mean (SD)	2.14 (0.561)	2.45 (0.553)	2.53 (0.658)	2.81 (0.704)	2.95 (1.480)	2.39 (0.654)
Geometric mean (GCV%)	2.06 (28.7)	2.37 (26.7)	2.44 (28.9)	2.72 (26.7)	2.76 (56.5)	2.29 (30.1)
Median (2.5%, 97.5%)	2.09 (1.02, 3.40)	2.45 (1.22, 3.57)	2.58 (1.34, 3.71)	2.92 (1.57, 4.10)	2.95 (1.95, 3.95)	2.35 (1.12, 3.73)
p-Value (ANOVA)						
vs. 7:00–10:00		0.0004	<0.0001	<0.0001		
vs. 11:00–14:00			0.3888	0.0039		
vs. 15:00–18:00				0.0211		

ANOVA, analysis of variance; BH_4_, 6R-L-erythro-5,6,7,8-tetrahydrobiopterin; GCV, geometric coefficient of variation; SD, standard deviation.

^a^ Data from >4 h were binned together due to limited data being available (n = 2).

#### Sex and race

Data from the 7:00–10:00 time block were used to investigate the potential differences in endogenous BH_4_ between sexes and races. This period contained the richest data, and endogenous BH_4_ was relatively stable, which minimized potential interference from diurnal change. Data from 161 out of 167 healthy participants were available in this time block for the analysis.

There were approximately equal numbers of male (*n* = 79) and female (*n* = 82) adult healthy volunteers included in this comparison (Figure 2B). BH_4_ concentrations overlapped substantially between male and female healthy volunteers (Figure 2B), though the geometric mean concentration was significantly higher in male participants (geometric mean, 2.18 ng/mL) than in female participants (1.95 ng/mL; Table 3).

**TABLE 3:** Endogenous BH_4_ concentrations by sex in healthy adult volunteers and adult patients with PKU.

Endogenous BH_4_ concentration, ng/mL	Healthy volunteers			
	Female (*n* = 82)	Male (*n* = 79)	Overall (*N* = 161)	p-Value (ANOVA)
Mean (SD)	2.03 (0.554)	2.25 (0.552)	2.14 (0.561)	0.0156
Geometric mean (CV%)	1.95 (30.1)	2.18 (26.2)	2.06 (28.7)	
Median (2.5%, 97.5%)	2.02 (0.98, 3.31)	2.18 (1.15, 3.53)	2.09 (1.02, 3.40)	

Endogenous BH_4_ concentration, ng/mL	Participants with PKU (≥18 years)			
	Female (*n* = 14)	Male (*n* = 10)	Overall (*N* = 24)	p-Value (ANOVA)
Mean (SD)	11.6 (3.8)	13.0 (6.2)	12.2 (4.9)	0.4972
Geometric mean (GCV%)	11.0 (36.2)	11.5 (61.1)	11.2 (46.3)	
Median (2.5%, 97.5%)	12.5 (6.05, 18.4)	13.5 (4.80, 21.2)	12.7 (4.95, 20.9)	

ANOVA, analysis of variance; BH_4_, 6R-L-erythro-5,6,7,8-tetrahydrobiopterin; CV, coefficient of variation; GCV, geometric coefficient of variation; PKU, phenylketonuria; SD, standard deviation.

Although the majority of the 161 participants available for this comparison were White (*n* = 105), there were enough Asian (*n* = 25) and Black or African American (*n* = 21) healthy volunteers to enable comparison of endogenous BH_4_ between races (Figure 2C). Asian participants had significantly higher levels of endogenous BH_4_ compared to White (2.33 ng/mL, p-value 0.0169), whereas levels were comparable between White (2.01 ng/mL) and Black or African American participants (2.07 ng/mL) (Figure 2C; Table 4).

**TABLE 4:** Endogenous BH_4_ concentrations according to races in healthy adult volunteers.

Endogenous BH_4_ concentration, ng/mL	Native American or Alaska native (*n* = 1)	Asian (*n* = 25)	Black or African American (*n* = 21)	White (*n* = 105)	Other (*n* = 9)
Mean (SD)		2.38 (0.52)	2.14 (0.57)	2.09 (0.55)	2.03 (0.76)
Geometric mean (GCV%)		2.33 (22.1)	2.07 (26.1)	2.01 (29.3)	1.90 (39.6)
Median (2.5%, 97.5%)	2.07 (2.07, 2.07)	2.40 (1.63, 3.43)	2.15 (1.37, 3.39)	2.07 (0.99, 3.16)	2.00 (1.12, 3.28)
p-Value (ANOVA)					
vs. Asian			0.1452	0.0169	0.1341

ANOVA, analysis of variance; BH_4_, 6R-L-erythro-5,6,7,8-tetrahydrobiopterin; GCV, geometric coefficient of variation; HV, healthy volunteer; SD, standard deviation.

#### Age

The same data from the 161 available healthy volunteers in the 7:00–10:00 time block were utilized to explore the effect of age on BH_4_ concentrations, which included participants from ages 18–56.6 years, with a median age of 33.4 years.

**FIGURE 3:**
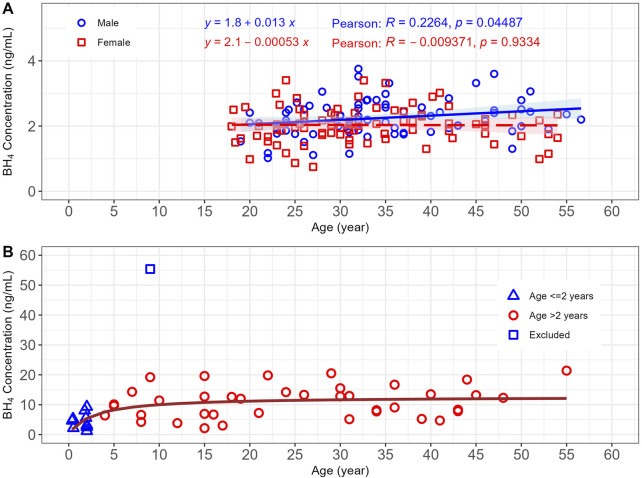
Change in endogenous BH_4_ concentration with age: **(A)** in healthy volunteers and **(B)** in participants with PKU. Shaded ribbon: 95% CI. BH_4_, *6R*-L-erythro-5,6,7,8-tetrahydrobiopterin; CI, confidence interval; PKU, phenylketonuria.

As illustrated in Figure 3A, there was essentially no change in endogenous BH_4_ concentration with age in both male and female participants. The slopes of linear regression for male and female healthy volunteers were within ±0.015 ng/mL per year of age. A Pearson correlation analysis was conducted to explore endogenous BH_4_ concentration with age. The correlation coefficients (r) for male and female participants were both within ±0.30, indicating the correlation was negligible between 18 and 57 years.

### Endogenous BH_4_ concentrations in participants with PBD and PKU

Predose and placebo treatment samples were collected from participants with PBD in a Phase 1b study and from participants with PKU in a placebo-controlled Phase 3 study and an open-label extension Phase 3 study (Supplementary Table S1). There were insufficient data across a wide enough time period to assess the circadian rhythm of BH_4_ concentrations in participants with either PBD or PKU. However, it was hypothesized that the pattern would resemble that observed in healthy volunteers. Thus, data collected in the 7:00–10:00 time block were utilized for the following analysis.

#### Participants with PBD

Data were available from a total of seven participants with PBD (all with 6-PTPS deficiency) with one BH_4_ measurement from each participant. As expected, the endogenous BH_4_ data were all BLQ (LLOQ 0.5 ng/mL; Table 1) because autosomal recessive 6-PTPS deficiency results in blocking of *de novo* BH_4_ biosynthesis (Opladen et al., 2020).

#### Participants with PKU

Predose and placebo treatment samples were collected from 62 participants with PKU (Table 1), and data in the 7:00–10:00 time block were available from 50 participants with PKU for the age and sex analysis. The graph in Figure 3B reveals that there is an obvious outlier, with a BH_4_ concentration of 55.4 ng/mL, which was significantly higher than the next highest observed BH_4_ concentration (21.4 ng/mL) and was therefore excluded from the analysis.

As there was a difference in endogenous BH_4_ concentration between male and female healthy volunteers, the sex difference was explored for participants with PKU. The analysis was limited to adult participants with PKU, as only this age group had an adequate number of participants (*n* = 24) for such analysis. No significant sex difference in endogenous BH_4_ concentration was observed for participants with PKU, and the ranges of observation highly overlapped, though the numeric value was slightly higher in male participants with PKU (Table 3).

Since endogenous BH_4_ concentrations were not distinguishable between male and female participants with PKU, and there were approximately equal numbers of male and female participants, the endogenous BH_4_ variations with age in participants with PKU were explored using combined male and female data (Figure 3B). Data collected from participants with PKU was not evenly distributed across ages. Considering the narrow age span, the age ≤2 years were over-represented (*n* = 3 for age <0.5 years and *n* = 7 for 1.5–2 years) compared with ages >2 years. There was a maximum of four participants for any 2-year span for those aged >2 years. BH_4_ concentrations in participants with PKU aged ≤2 years (*n* = 10) were significantly lower than in those aged >2 years. The geometric mean (95% CI) of endogenous BH_4_ for PKU patients of age ≤2 years and >2 years were 3.80 (1.40–9.02) and 9.63 (2.98–20.5), respectively (Supplementary Table S3). A simple E_max_ equation [Endogenous BH_4_ concentration = E_max_*Age/(AM50+Age), where E_max_ was the maximum endogenous BH_4_ concentration, AM50 was the age that endogenous reached 50% of the maximum] best described the relationship between endogenous BH_4_ concentration and age (Figure 3B). The E_max_ and AM50 values were 12.69 ng/mL and 2.70 years, respectively. There was essentially no change in central trend of endogenous BH_4_ concentration in participants with PKU aged >2 years. This was confirmed with the Pearson correlation analysis for data in the group aged >2 years, which yielded a correlation coefficient (R) of 0.2265 and a p-value of 0.1656 (Supplementary Figure S1).

Hence, only data from participants with PKU aged >2 years (*n* = 39) were included to assess the population difference in those with different disease statuses (Figure 2D). The endogenous BH_4_ concentration was significantly higher in participants with PKU (9.63 ng/mL) than in healthy volunteers (2.06 ng/mL, p-value <0.0001) (Table 5). It was clear that there was no overlapping of participants with PBD with healthy volunteers or participants with PKU, and minimal overlapping of participants with PKU or healthy volunteers.

**TABLE 5:** Endogenous BH_4_ concentrations according to diseases.

Endogenous BH_4_ concentration, ng/mL	HV (*n* = 161)	PBD (*n* = 7)	PKU (aged >2 years) (*n* = 39)	Overall (*N* = 207)
Mean (SD)	2.14 (0.56)	0	11.0 (5.27)	3.74 (4.23)
Geometric mean (GCV%)	2.06 (28.7)		9.63 (60.7)	
Median (2.5%, 97.5%)	2.09 (1.02, 3.40)	0 (0, 0)	11.4 (2.98, 20.5)	2.19 (0, 18.1)
p-Value (ANOVA)				
vs. HV		<0.0001	<0.0001	

ANOVA, analysis of variance; BH_4_, 6R-L-erythro-5,6,7,8-tetrahydrobiopterin; GCV, geometric coefficient of variation; HV, healthy volunteer; PBD, primary tetrahydrobiopterin deficiency; PKU, phenylketonuria; SD, standard deviation.

## Discussion

In healthy volunteers, a circadian rhythm of BH_4_ concentrations was identified. BH_4_ concentration was the lowest in the morning between 7:00 and 10:59 (geometric mean 2.06 ng/mL) and the highest late at night between 21:00 and 22:59 (2.72 ng/mL). However, this variation (0.66 ng/mL) was comparable to the inter-subject variability (0.561 ng/mL for the standard deviation in the 7:00–10:00 time block). Endogenous BH_4_ was relatively stable in the 7:00–10:00 time block, and this unique characteristic makes it a convenient period to evaluate endogenous BH_4_ concentrations according to different participant characteristics, including age, sex, race, and disease status.

In healthy adult volunteers, there was a significant difference in endogenous plasma BH_4_ concentration between male (2.18 ng/mL) and female participants (1.95 ng/mL). However, this difference lacks clinical relevance, as it falls below the inter-subject variability. Similarly, a significant difference between Asian (2.33 ng/mL) and White (2.01 ng/mL) was noted. Nevertheless, this difference does not translate into clinical significance owing to extensive inter-subject variability.

Endogenous BH_4_ concentrations were significantly elevated in participants with PKU (9.63 ng/mL for those aged >2 years) compared to healthy adults (2.06 ng/mL). Moreover, BH_4_ concentrations in both participants with PKU and healthy volunteers were substantially higher than the essentially zero concentration observed in participants with PBD.

In participants with PKU with age above 2 years, the majority of data were collected between 7:00 and 10:59 (*n* = 77, 70.0%) and the remaining were collected between 11:00 and 16:59 (*n* = 33, 30.0%). There was insufficient data for a thorough analysis to detect the diurnal variation in participants with PKU. However, an exploratory graphic analysis indicated that the diurnal variation would be negligible from 7:00 to 16:59 compared to the inter-subject variability, if there were any (Supplementary Figure S2). The difference in endogenous plasma BH_4_ between male and female participants with PKU was not significant; this was attributed to the inter-subject variability, which was even larger than that observed in healthy volunteers. Since endogenous BH_4_ concentrations were essentially zero in participants with PBD, no correlation with circadian rhythm or sex was expected.

All seven participants with PBD were affected by an autosomal recessive deficiency in 6-PTPS, and their endogenous BH_4_ concentrations were BLQ (LLOQ 0.5 ng/mL). PBD arises from variants in five genes encoding enzymes responsible for the biosynthesis and regeneration of BH_4_, including guanosine triphosphate cyclohydrolase I (GTPCH-I), sepiapterin reductase (SR), 6-PTPS, pterin-4-alpha-carbinolamine dehydratase (PCD), and dihydropteridine reductase (DHPR) (Opladen et al., 2020). GTPCH-I, 6-PTPS, and SR are important enzymes for *de novo* biosynthesis of BH_4_, while PCD and DHPR are key enzymes for BH_4_ regeneration (Werner et al., 2011). Reduced enzyme activity of 6-PTPS could hinder *de novo* biosynthesis of BH_4_, leading to significantly lower plasma BH_4_, which was essentially zero, as observed in this study.

It has long been understood that BH_4_ deficiency results from the aforementioned enzyme deficiencies. Most PBDs are routinely diagnosed by the presence of HPA during newborn screening and confirmed following pterin analysis (neopterin, biopterin, isoxanthopterin, and primapterin) in a dried blood spot or urine analysis. Additionally, patients with PBD exhibit significant reductions in neurotransmitters such as dopamine, serotonin, and norepinephrine. Plasma BH_4_ concentration has not been routinely used as a diagnostic tool. Results from this study indicate that plasma BH_4_ concentrations in participants with 6-PTPS deficiency can be distinctly differentiated from those in healthy volunteers and those with other HPA conditions, such as PKU.

PKU is a metabolic disorder caused by a defect or deficiency in PAH due to mutant variants in the corresponding *PAH* gene. By 2020, more than 950 PAH variants and 3,659 genotypes have been identified (Hillert et al., 2020). PAH catalyzes the hydroxylation of phenylalanine to form tyrosine. A deficiency in PAH results in elevated blood Phe concentration known as HPA (Kaufman, 1993; Blau et al., 2010; van Spronsen et al., 2021). Human PAH is a cytosolic enzyme that exists in solution as a pH-dependent equilibrium between functional tetramers and dimers (Martinez et al., 1995). Phe activates PAH by binding to the enzyme and shifts the configuration from dimer-dominant to tetramer-dominant (Martinez et al., 1995). BH_4_ acts as an essential cofactor for PAH (Bailey et al., 1993). BH_4_ binds to the catalytic site of PAH to block the conformation change and hence stabilize the enzyme (Kaufman, 1993; Pey et al., 2004; Heintz et al., 2013). During Phe hydroxylation, BH_4_ is oxidized, transferring two electrons to the enzyme complex before leaving the catalytic site as 4α-hydroxy-tetrahydrobiopterin (Werner et al., 2011). Higher blood Phe concentration necessitates higher BH_4_ concentration to sustain the PAH activity (Staudigl et al., 2011; Heintz et al., 2013). Consequently, it is unsurprising to observe higher endogenous BH_4_ concentrations in patients with PKU compared to healthy individuals. The elevated BH_4_ concentrations may serve to prevent degradation of mutant PAH and maintain PAH activity in the presence of higher Phe concentrations.

Beyond its role in PAH, BH_4_ acts as a cofactor for other aromatic amino acid hydroxylases, including TH, TPH, AGMO, and NOS. Abnormal endogenous BH_4_ concentration is associated with diseases involving these enzymatic pathways. BH_4_ is crucial for the coupling of endothelial NOS (eNOS), which stabilizes eNOS and regulates nitric oxide (NO) production. A decrease in BH_4_ could lead to eNOS dysfunction, a reduction in NO production, and an increase in reactive oxygen species, which are linked to various diseases, such as cardiovascular disease, diabetes, autism, and cancer (Kim and Han, 2020; Goncalves et al., 2021). Reduced BH_4_ in the brain could impair TH activity, the enzyme involved in dopamine synthesis, and has been associated with idiopathic Parkinson’s disease (Eichwald et al., 2023). Furthermore, deficiency in BH_4_ bioavailability is linked to distinct diseases, such as Alzheimer’s disease, Fabry disease, and certain mitochondrial diseases (Eichwald et al., 2023). Establishing a reference range for plasma BH_4_ concentrations across various diseases could greatly aid in diagnostic processes.

In healthy adult volunteers and participants with PKU >2 years of age, endogenous BH_4_ concentrations had no correlation with age. However, lower plasma BH_4_ concentrations were noted in participants with PKU aged ≤2 years. The connection between this observation and the pathology of PKU in this age group is not yet fully understood; further research is warranted to elucidate the underlying mechanisms.

In a recent publication, endogenous BH_4_ concentrations from heathy volunteers and patients were summarized (Wang et al., 2024). The mean BH_4_ concentration calculated as a weighted average [(n1*mean1 + n2*mean2 + …)/(n1+n2+ …), where n1 and mean1 referred to the number of subjects and mean BH_4_ reported in study 1, …] for adult healthy volunteers was 2.94 ng/mL from 11 studies cited in the report from 522 individuals which included 253 Chinese (Wang et al., 2024). The mean BH_4_ was 2.48 ng/mL after excluding Chinese healthy volunteers. One study involving 38 healthy Chinese aged 18–65 years reported a rapid reduction in plasma BH_4_ with age, from 6.80 ng/mL at 18 years to 0.66 ng/mL at 65 years with the mean (SD) BH_4_ 3.09 (1.43) ng/mL (Yuan et al., 2018). In the study, blood samples were stabilized with 0.2% DTE (v:v) immediately after collection. BH_4_ was measured using HPLC-MS after derivatization with benzoyl chloride following acetonitrile protein precipitation to enhance the sensitivity (LLOQ 0.05 ng/mL). The extent of endogenous BH_4_ reduction with age in adults was steep (90% reduction across 47 years during adulthood from 18 to 65 years), which seems implausible considering the role of BH_4_ as a cofactor for multiple essential enzymes and the necessity of stable BH_4_ required for normal functionality. A study from the same laboratory used an improved HPLC-MS method, based on Yuan et al. (2018) with benzyl chloride derivatization followed by cold-induced phase separation for 5 min at −30 °C, to measure endogenous BH_4_ from 215 healthy Chinese aged 13 to 148 (it was suspected the top age was entered as a mistake, the highest age second to 148 was 91) (Wang et al., 2024). Compared to the first study reported from the lab (Yuan et al., 2018), mean (SD) plasma BH_4_ concentration from this recent study was higher at 3.51 (0.94) ng/mL. However, the trend of BH_4_ concentration reduction with age was much flatter. From age 22 to 91 (after excluding subjects of age 13 and 148, which were far away from rest of subjects), the mean BH_4_ from linear regression based on the reported data dropped from 4.06 to 2.68 ng/mL, a decrease of 34% for an age span of 69 years from 22 to 91. In our study, mean BH_4_ from adult healthy volunteers in the morning between 7:00 and 10:59 was (2.14 [0.56] ng/mL) and it was independent of age. This value is slightly lower than the mean value excluding Chinese from historical studies (2.48 ng/mL) and much lower than the values reported for Chinese (Yuan et al., 2018; Wang et al., 2024). The discrepancy can be multifactorial. First, results from this study indicated that BH_4_ concentrations were lower in the morning and higher in the afternoon and evening. It is unknown the time of day blood samples were collected in those historical studies. Second, there is an ethnic difference in endogenous BH_4_ with the mean concentration higher in Asian and lower in Whites. The ethnic factor should be considered when comparing endogenous BH_4_ between races. Third, there is no cross validation between methods and laboratories. As discussed in Wang et al. (2024) report that timely addition of anti-oxidation immediately post blood collection is critical for accurate measurement of plasma BH_4_. DTT, DTE, and ascorbic acid are frequently used reagents to stabilize BH_4_ (Fiege et al., 2004; Fekkes and Voskuilen-Kooijman, 2007; Kaushik et al., 2024). In this study, blood samples were stabilized with 1% ascorbic acid within 15 min of blood collection and the supernatant from centrifugation after protein precipitation (containing DTE, DETPC in acetonitrile/water) were dried under nitrogen in approximately 45 min at 40 °C (Kaushik et al., 2024). The variations in stabilizing reagents used, and timing of the anti-oxidation reagent addition, as well as the following sample processing procedures are not fully understood at this time. The bioanalytical method used in this study has been used in 10 clinical trials to measure plasma BH_4_ concentrations in over 6300 blood samples from over 600 individuals. All bioanalytical runs have consistently passed the rigorous validated criteria, and the 10% incurred sample reanalysis passed the acceptance criteria as well. The consistency of reliable performance of this validated bioanalytical method warranted the reliability of results from this study. Additionally, there were large intersubject variability for BH_4_. Sufficient sample size is required to obtain a true mean value from the population. This reflected in the two studies conducted in Chinese healthy volunteers. A steep age dependent BH_4_ decrease was observed from Yuan et al. (2018) study, which included 38 participants and 1 blood sample per subject. The age dependent trend was much flatter in the recent study reported by Wang et al. (2024), which included 215 participants. In this study, data were obtained from 167 participants and 1058 blood samples in the morning between 7:00 and 10:59. The comparable value from this study and mean value from previous studies in healthy volunteers and the consistency between this study and multiple prior reports involving patients with PKU across ages 0–50 years, combined with the abundancy of data from this study, suggest that our findings are reliable–BH_4_ concentration remains stable in healthy adults regardless of age. The geometric mean (95% percentile) endogenous BH_4_ concentration of healthy adults is 2.06 (1.02, 3.40) ng/mL.

Although endogenous BH_4_ concentrations in participants with PKU were not directly reported in previous studies, the value could be inferred from the baseline BH_4_ concentrations of population pharmacokinetic models. The baseline BH_4_ concentrations were 13.5 ng/mL for participants with PKU aged ≥8 years (*n* = 78) (Feillet et al., 2008), 12.6 ng/mL for participants with PKU aged <4 years (*n* = 52) (Muntau et al., 2017), and 16.6 ng/mL for participants with PKU aged 0–50 years (*n* = 156), wherein 80 participants were aged <8 years (Qi et al., 2015). These findings align with our observation of a mean (SD) BH_4_ concentration of 11.0 (5.27) ng/mL for participants with PKU aged >2 years (*n* = 39), and geometric mean (95% percentile) of 9.63 (2.98, 20.5) ng/mL.

In one report, a rapid decrease in BH_4_ concentration in cerebrospinal fluid (CSF) with age was reported for newborns (0–0.33 years, *n* = 12), followed by relatively stable concentrations from ages ≥0.34–20 years (*n* = 61) in participants with neurologic disease (Hyland et al., 1993). A similar pattern was reported for BH_4_ in CSF from 99 participants with various neurologic disorders ranging from 0 to 42 years old, in which BH_4_ concentrations were stable at least across ages 1.1–20 years (Guibal et al., 2014). In both studies, CSF samples were immediately frozen at −80 °C after collection without addition of any stabilizing reagent. The indirect chemical oxidation method coupled with HPLC-FD was used to determine BH_4_ concentrations. The stability of BH_4_ in CSF samples was not reported in either study.

Although lower than adult endogenous BH_4_ concentrations were noted in PKU patients with age ≤2 years in our study, it cannot be definitively concluded that this observation is not coincidental, given the small sample size of participants with PKU within this age range (*n* = 10).

## Conclusion

A clear circadian rhythm of BH_4_ concentrations was observed in healthy adults, with the lowest concentration occurring in the morning (7:00–10:59) and gradually increasing throughout the day to the highest concentration in the late evening (21:00–22:59). Additionally, endogenous BH_4_ concentrations were higher in male than in female participants, and higher in Asians than in other races in healthy adults. However, the magnitude of all such fluctuations was small and comparable to inter-subject variability suggesting a lack of clinical relevance. No correlation was found between endogenous BH_4_ levels and age in healthy adults.

Endogenous plasma BH_4_ concentrations were found to be relatively stable between 7:00 and 10:00, providing a suitable time window for BH_4_ sample collection to minimize measurement variation due to diurnal effects.

Furthermore, endogenous plasma BH_4_ concentration ranges differ significantly between participants with PBD, participants with PKU, and healthy volunteers. This difference could be utilized as a diagnostic tool.

## Data Availability

The raw data supporting the conclusions of this article will be made available by the authors, upon reasonable request.

## References

[B1] Bailey S. W. Boerth S. R. Dillard S. B. Ayling J. E. (1993). The mechanism of cofactor regeneration during phenylalanine hydroxylation. Adv. Exp. Med. Biol. 338, 47–54. 10.1007/978-1-4615-2960-6_9 8304161

[B2] Blair J. A. Pearson A. J. (1973). A kinetic study of the automation of terrahydrobiopterin. Tetrahedron Lett. 14 (3), 203–204. 10.1016/S0040-4039(01)95618-7

[B3] Blau N. (2013). Sapropterin dihydrochloride for the treatment of hyperphenylalaninemias. Expert Opin. Drug Metab. Toxicol. 9 (9), 1207–1218. 10.1517/17425255.2013.804064 23705856

[B4] Blau N. van Spronsen F. J. Levy H. L. (2010). Phenylketonuria. Lancet 376 (9750), 1417–1427. 10.1016/S0140-6736(10)60961-0 20971365

[B5] Boulghobra A. Bonose M. Alhajji E. Pallandre A. Flamand-Roze E. Baudin B. (2023). Autoxidation kinetics of tetrahydrobiopterin-giving quinonoid dihydrobiopterin the consideration it deserves. Molecules 28 (3), 1267. 10.3390/molecules28031267 36770933 PMC9921404

[B6] Buglak A. A. Telegina T. A. Vechtomova Y. L. Kritsky M. S. (2021). Autoxidation and photooxidation of tetrahydrobiopterin: a theoretical study. Free Radic. Res. 55 (5), 499–509. 10.1080/10715762.2020.1860213 33283562

[B7] Eichwald T. da Silva L. d.B. Staats Pires A. C. Niero L. Schnorrenberger E. Filho C. C. (2023). Tetrahydrobiopterin: beyond its traditional role as a cofactor. Antioxidants 12 (5), 1037. 10.3390/antiox12051037 37237903 PMC10215290

[B8] Fanet H. Capuron L. Castanon N. Calon F. Vancassel S. (2021). Tetrahydrobioterin (BH4) pathway: from metabolism to neuropsychiatry. Curr. Neuropharmacol. 19 (5), 591–609. 10.2174/1570159X18666200729103529 32744952 PMC8573752

[B9] Feillet F. Clarke L. Meli C. Lipson M. Morris A. A. Harmatz P. (2008). Pharmacokinetics of sapropterin in patients with phenylketonuria. Clin. Pharmacokinet. 47 (12), 817–825. 10.2165/0003088-200847120-00006 19026037

[B10] Fekkes D. Voskuilen-Kooijman A. (2007). Quantitation of total biopterin and tetrahydrobiopterin in plasma. Clin. Biochem. 40 (5-6), 411–413. 10.1016/j.clinbiochem.2006.12.001 17291474

[B11] Fiege B. Ballhausen D. Kierat L. Leimbacher W. Goriounov D. Schircks B. (2004). Plasma tetrahydrobiopterin and its pharmacokinetic following oral administration. Mol. Genet. Metab. 81 (1), 45–51. 10.1016/j.ymgme.2003.09.014 14728990

[B12] Fismen L. Eide T. Djurhuus R. Svardal A. M. (2012). Simultaneous quantification of tetrahydrobiopterin, dihydrobiopterin, and biopterin by liquid chromatography coupled electrospray tandem mass spectrometry. Anal. Biochem. 430 (2), 163–170. 10.1016/j.ab.2012.08.019 22940649

[B13] Fukushima T. Nixon J. C. (1980). Analysis of reduced forms of biopterin in biological tissues and fluids. Anal. Biochem. 102 (1), 176–188. 10.1016/0003-2697(80)90336-x 7356152

[B14] Gao L. Kaushik D. Ingalls K. Milner S. Smith N. Kong R. (2024a). Clinical assessment of breast cancer resistance protein (BCRP)-mediated drug-drug interactions of sepiapterin with curcumin and rosuvastatin in healthy volunteers. Drugs R. D. 24, 477–487. 10.1007/s40268-024-00488-0 39316278 PMC11455768

[B15] Gao L. Kaushik D. Ingalls K. Smith N. Kong R. (2024b). A phase 1 study to assess the pharmacokinetics, food effect, safety, and tolerability of sepiapterin in healthy Japanese and non-japanese participants. Pharm. (Basel) 17 (11), 1411. 10.3390/ph17111411 39598323 PMC11597218

[B16] Gao L. Kaushik D. Xia Y. Ingalls K. Milner S. Smith N. (2024c). Relative oral bioavailability and food effects of two sepiapterin formulations in healthy participants. Clin. Pharmacol. Drug Dev. 13 (5), 506–516. 10.1002/cpdd.1363 38156759

[B17] Goncalves D. A. Jasiulionis M. G. Melo F. H. M. (2021). The role of the BH4 cofactor in nitric oxide synthase activity and cancer progression: two sides of the same coin. Int. J. Mol. Sci. 22 (17), 9546. 10.3390/ijms22179546 34502450 PMC8431490

[B18] Guibal P. Leveque N. Doummar D. Giraud N. Roze E. Rodriguez D. (2014). Simultaneous determination of all forms of biopterin and neopterin in cerebrospinal fluid. ACS Chem. Neurosci. 5 (7), 533–541. 10.1021/cn4001928 24650440 PMC4102970

[B19] Guibal P. Lo A. Maitre P. Moussa F. (2017). Pterin determination in cerebrospinal fluid: state of the art. Pteridines 28 (2), 83–89. 10.1515/pterid-2017-0001

[B20] Heintz C. Cotton R. G. Blau N. (2013). Tetrahydrobiopterin, its mode of action on phenylalanine hydroxylase, and importance of genotypes for pharmacological therapy of phenylketonuria. Hum. Mutat. 34 (7), 927–936. 10.1002/humu.22320 23559577

[B21] Hillert A. Anikster Y. Belanger-Quintana A. Burlina A. Burton B. K. Carducci C. (2020). The genetic landscape and epidemiology of phenylketonuria. Am. J. Hum. Genet. 107 (2), 234–250. 10.1016/j.ajhg.2020.06.006 32668217 PMC7413859

[B22] Hyland K. (1985). 'Estimation of tetrahydro, dihydro and fully oxidised pterins by high-performance liquid chromatography using sequential electrochemical and fluorometric detection. J. Chromatogr. 343 (1), 35–41. 10.1016/s0378-4347(00)84565-x 4066860

[B23] Hyland K. Surtees R. A. Heales S. J. Bowron A. Howells D. W. Smith I. (1993). Cerebrospinal fluid concentrations of pterins and metabolites of serotonin and dopamine in a pediatric reference population. Pediatr. Res. 34 (1), 10–14. 10.1203/00006450-199307000-00003 7689195

[B24] Jung-Kc K. Tristan-Noguero A. Altankhuyag A. Pinol Belenguer D. Prestegard K. S. Fernandez-Carasa I. (2024). Tetrahydrobiopterin (BH(4)) treatment stabilizes tyrosine hydroxylase: rescue of tyrosine hydroxylase deficiency phenotypes in human neurons and in a knock-in mouse model. J. Inherit. Metab. Dis. 47 (3), 494–508. 10.1002/jimd.12702 38196161

[B25] Kaufman S. (1993). The phenylalanine hydroxylating system. Adv. Enzymol. Relat. Areas Mol. Biol. 67, 77–264. 10.1002/9780470123133.ch2 8322620

[B26] Kaushik D. Gao L. Yuan K. Tang B. Ronald K. (2024). LC-MS/MS methods for direct measurement of sepiapterin and tetrahydrobiopterin in human plasma and clinical applications. Bioanalysis 16 (2), 75–89. 10.4155/bio-2023-0144 38099558

[B27] Kim H. K. Han J. (2020). Tetrahydrobiopterin in energy metabolism and metabolic diseases. Pharmacol. Res. 157, 104827. 10.1016/j.phrs.2020.104827 32348841

[B28] Kim H. R. Kim T. H. Hong S. H. Kim H. G. (2012). Direct detection of tetrahydrobiopterin (BH4) and dopamine in rat brain using liquid chromatography coupled electrospray tandem mass spectrometry. Biochem. Biophys. Res. Commun. 419 (4), 632–637. 10.1016/j.bbrc.2012.02.064 22382017

[B29] Komleva P. D. Deeb R. Terentieva E. I. Kulikov A. V. (2025). Comparison of the effects of tetrahydrobiopterine, L-Tryptophan, and iron ions on the thermal stability of wild type and P447R mutant tryptophan hydroxylase 2. Bull. Exp. Biol. Med. 178 (4), 447–452. 10.1007/s10517-025-06354-6 40140132

[B30] Martinez A. Knappskog P. M. Olafsdottir S. Doskeland A. P. Eiken H. G. Svebak R. M. (1995). Expression of recombinant human phenylalanine hydroxylase as fusion protein in *Escherichia coli* circumvents proteolytic degradation by host cell proteases. Isolation and characterization of the wild-type enzyme. Biochem. J. 306 (Pt 2), 589–597. 10.1042/bj3060589 7887915 PMC1136558

[B31] Mortensen A. Lykkesfeldt J. (2013). Kinetics of acid-induced degradation of tetra- and dihydrobiopterin in relation to their relevance as biomarkers of endothelial function. Biomarkers 18 (1), 55–62. 10.3109/1354750X.2012.730552 23066920

[B32] Muntau A. C. Burlina A. Eyskens F. Freisinger P. De Laet C. Leuzzi V. (2017). Efficacy, safety and population pharmacokinetics of sapropterin in PKU patients <4 years: results from the SPARK open-label, multicentre, randomized phase IIIb trial. Orphanet J. Rare Dis. 12 (1), 47. 10.1186/s13023-017-0600-x 28274234 PMC5343543

[B33] Muntau A. C. Longo N. Ezgu F. Schwartz I. V. D. Lah M. Bratkovic D. (2024). Effects of oral sepiapterin on blood phe concentration in a broad range of patients with phenylketonuria (APHENITY): results of an international, phase 3, randomised, double-blind, placebo-controlled trial. Lancet 404 (10460), 1333–1345. 10.1016/S0140-6736(24)01556-3 39368841

[B34] Opladen T. Lopez-Laso E. Cortes-Saladelafont E. Pearson T. S. Sivri H. S. Yildiz Y. (2020). Consensus guideline for the diagnosis and treatment of tetrahydrobiopterin (BH(4)) deficiencies. Orphanet J. Rare Dis. 15 (1), 126. 10.1186/s13023-020-01379-8 32456656 PMC7251883

[B35] Pey A. L. Perez B. Desviat L. R. Martinez M. A. Aguado C. Erlandsen H. (2004). Mechanisms underlying responsiveness to tetrahydrobiopterin in mild phenylketonuria mutations. Hum. Mutat. 24 (5), 388–399. 10.1002/humu.20097 15459954

[B36] Qi Y. Mould D. R. Zhou H. Merilainen M. Musson D. G. (2015). A prospective population pharmacokinetic analysis of sapropterin dihydrochloride in infants and young children with phenylketonuria. Clin. Pharmacokinet. 54 (2), 195–207. 10.1007/s40262-014-0196-4 25338975 PMC4306193

[B37] Smith N. Longo N. Levert K. Hyland K. Blau N. (2019). Phase I clinical evaluation of CNSA-001 (sepiapterin), a novel pharmacological treatment for phenylketonuria and tetrahydrobiopterin deficiencies, in healthy volunteers. Mol. Genet. Metab. 126 (4), 406–412. 10.1016/j.ymgme.2019.02.001 30922814

[B38] Staudigl M. Gersting S. W. Danecka M. K. Messing D. D. Woidy M. Pinkas D. (2011). The interplay between genotype, metabolic state and cofactor treatment governs phenylalanine hydroxylase function and drug response. Hum. Mol. Genet. 20 (13), 2628–2641. 10.1093/hmg/ddr165 21527427

[B39] Telegina T. A. Lyudnikova T. A. Buglak A. A. Vechtomova Y. L. Biryukov M. V. Demin V. V. (2018). Transformation of 6-tetrahydrobiopterin in aqueous solutions under UV-irradiation. J. Photochem. Photobiol. A Chem. 354, 155–162. 10.1016/j.jphotochem.2017.07.029

[B40] Thony B. Ding Z. Martinez A. (2004). Tetrahydrobiopterin protects phenylalanine hydroxylase activity *in vivo:* implications for tetrahydrobiopterin-responsive hyperphenylalaninemia. FEBS Lett. 577 (3), 507–511. 10.1016/j.febslet.2004.10.056 15556637

[B41] Thony B. Calvo A. C. Scherer T. Svebak R. M. Haavik J. Blau N. (2008). Tetrahydrobiopterin shows chaperone activity for tyrosine hydroxylase. J. Neurochem. 106 (2), 672–681. 10.1111/j.1471-4159.2008.05423.x 18419768

[B42] van Spronsen F. J. Blau N. Harding C. Burlina A. Longo N. Bosch A. M. (2021). Phenylketonuria. Nat. Rev. Dis. Prim. 7 (1), 36. 10.1038/s41572-021-00267-0 34017006 PMC8591558

[B43] van Spronsen F. Peters H. Margvelashvili L. Agladze D. Schwartz I. Gizewska M. (2025). Effect of long-term sepiapterin treatment on dietary phenylalanine tolerance in patients with phenylketonuria: interim results from the phase 3 APHENITY extension study. Genet. Med. Accept. Publ. 10.1016/j.gim.2026.101683 41537382

[B44] Wang H. B. Xiao X. Dai W. Cui Y. Li W. M. Peng R. (2024). Dispel some mist on circulating biopterins: measurement, physiological interval and pathophysiological implication. Metabolomics 20 (4), 74. 10.1007/s11306-024-02137-8 38980520

[B45] Werner E. R. (2013). Three classes of tetrahydrobiopterin-dependent enzymes. Pteridines 24 (1), 7–11. 10.1515/pterid-2013-0003

[B46] Werner E. R. Blau N. Thony B. (2011). Tetrahydrobiopterin: biochemistry and pathophysiology. Biochem. J. 438 (3), 397–414. 10.1042/BJ20110293 21867484

[B47] Werner-Felmayer G. Golderer G. Werner E. R. (2002). Tetrahydrobiopterin biosynthesis, utilization and pharmacological effects. Curr. Drug Metab. 3 (2), 159–173. 10.2174/1389200024605073 12003348

[B48] Xu X. S. Dunne A. Kimko H. Nandy P. Vermeulen A. (2011). Impact of low percentage of data below the quantification limit on parameter estimates of pharmacokinetic models. J. Pharmacokinet. Pharmacodyn. 38 (4), 423–432. 10.1007/s10928-011-9201-9 21626437

[B49] Yuan T. F. Huang H. Q. Gao L. Wang S. T. Li Y. (2018). A novel and reliable method for tetrahydrobiopterin quantification: benzoyl chloride derivatization coupled with liquid chromatography-tandem mass spectrometry analysis. Free Radic. Biol. Med. 118, 119–125. 10.1016/j.freeradbiomed.2018.02.035 29501564

[B50] Zhao Y. Cao J. Chen Y. S. Zhu Y. Patrick C. Chien B. (2009). Detection of tetrahydrobiopterin by LC-MS/MS in plasma from multiple species. Bioanalysis 1 (5), 895–903. 10.4155/bio.09.77 21083061

