# Correction: Endogenous tetrahydrobiopterin in humans: circadian rhythm, sex, race, age, and disease status

**DOI:** 10.3389/fphar.2025.1764401

**Published:** 2025-12-19

**Authors:** Lan Gao, Neil Smith, Ronald Kong

**Affiliations:** PTC Therapeutics, Inc., Warren, NJ, United States

**Keywords:** 5,6,7,8-tetrahydrobiopterin (BH_4_), circadian rhythm, sex, race, age, phenylketonuria (PKU), primary tetrahydrobiopterin deficiency (PBD)

E_max_ was erroneously written as E_max*Age_.

A correction has been made to the section **Results**, Endogenous BH4 concentrations in participants with PBD and PKU, Participants with PKU, Paragraph Number 3:

“The E_max_ and AM50 values were 12.69 ng/mL and 2.70 years, respectively. There was essentially no change in central trend of endogenous BH4 concentration in participants with PKU aged >2 years. This was confirmed with the Pearson correlation analysis for data in the group aged >2 years, which yielded a correlation coefficient (R) of 0.2265 and a p-value of 0.1656 (Supplementary Figure S1).”

The original article has been updated.

