# Supplementary material for: Endogenous tetrahydrobiopterin in humans: circadian rhythm, sex, race, age, and disease status
Source: Front Pharmacol. 2025 Dec 5;16:1701617. doi: 10.3389/fphar.2025.1701617 (PMC12757610; doi:10.3389/fphar.2025.1701617)
Supplement: Supplementary file 1 [file Supplementaryfile1.docx]

Supplementary Material

Endogenous tetrahydrobiopterin (BH_4_) in human: circadian rhythm, sex, race, age, and disease status

**Table S1. Summary of Clinical Studies Included in the Analysis**

| **Study** | **Population** | **Description** | **PK Sampling (Endogenous BH_4_)** | **Placebo Treatment** | **Multiple Period** | **Washout (day)** | **Reference** |
| --- | --- | --- | --- | --- | --- | --- | --- |
| PKU-001 | HV (n=80),  Age ≥18 years | SAD, MAD, and food effect | Day 1 predose SAD/MAD | Yes (during SAD study) | No |  | Smith 2019 |
| PTC923-MD-005-HV | HV (n=34),  Age ≥18 years | Part A: relative bioavailability  Part B: food effect  2-sequence, 4-period cross over single dose for both parts | Day-1 and Day 1 predose | No | 4 periods | 3 | Gao 2024c |
| PTC923-MD-007-JPN | HV (n=60),  Age ≥18 years | Single dose  2-sequence 2-period cross over food effect for Japanese at 40 mg/kg only | Day-1 and Day 1 predose | No | 2 periods | 3 | Gao 2024b |
| PTC923-DDI-101-HV | HV (n=29),  Age ≥18 years | Single dose, fixed sequence 4-period cross over drug interaction study | Day 1 Predose | No | 4 periods | 4 | Gao 2024a |
| PTC923-TQT-102-HV | HV (n=32),  Age ≥18 years | 12-sequence 4-period cross over thorough QT study | Day 1 predose  (-75, -60, and -45 min) | Yes | 4 periods | 4 |  |
| PBD-001 | PBD (n=8),  Age 1 to 20 years | 2-period 7-day treatment Phase 1b study | Day 1 predose | No | 2 periods | 3 (±1) |  |
| PTC923-MD-003-PKU | PKU (n=157),  All ages | Part 1: 14-day fixed dose treatment Part 2: placebo-controlled double-blind treatment | Part 1: Day 1 predose, Part 2 Day 1 predose | Yes, Part 2 | 1 period for Part 1  3 periods for Part 2 | 14 to 21 | Muntau 2024 |
| PTC923-MD-004-PKU | PKU, Ongoing, n=65, All Ages,  Data cut by 30June2024 | Open label treatment of PKU patients with sepiapterin | Day 1 predose | No | No | No | van Spronsen 2025 |

**Table S2. Endogenous BH_4_ Measurements in Adult HV by Hour**

| **24 Hour Clock** | 1 (N=1) | 7 (N=16) | 8 (N=55) | 9 (N=128) | 10 (N=57) | 11 (N=44) | 12 (N=39) | 13 (N=41) | 14 (N=47) | 15 (N=61) |
| --- | --- | --- | --- | --- | --- | --- | --- | --- | --- | --- |
|  |  |  |  |  |  |  |  |  |  |  |
| Mean (SD) |  | 2.51 (0.990) | 2.29 (0.689) | 2.13 (0.558) | 2.25 (0.647) | 2.49 (0.553) | 2.59 (0.594) | 2.56 (0.499) | 2.53 (0.680) | 2.68 (0.584) |
| Geometric Mean (GCV%) |  | 2.35 (37.0%) | 2.22 (25.0%) | 2.06 (28.0%) | 2.15 (32.3%) | 2.43 (22.4%) | 2.52 (23.5%) | 2.52 (19.2%) | 2.43 (30.7%) | 2.62 (23.1%) |
| Median [95%] | 1.90  [1.90, 1.90] | 2.19  [1.48, 4.61] | 2.13  [1.60, 4.56] | 2.09  [1.12, 3.39] | 2.18  [1.11, 3.48] | 2.45  [1.61, 3.62] | 2.52  [1.64, 3.88] | 2.53  [1.90, 3.70] | 2.46  [1.06, 3.65] | 2.67  [1.57, 3.82] |

| **24 Hour Clock** | 16 (N=33) | 17 (N=34) | 18 (N=21) | 19 (N=18) | 20 (N=17) | 21 (N=25) | 22 (N=13) | 23 (N=1) | Overall (N=651) |
| --- | --- | --- | --- | --- | --- | --- | --- | --- | --- |
|  |  |  |  |  |  |  |  |  |  |
| Mean (SD) | 2.53 (0.846) | 2.57 (0.574) | 2.72 (0.652) | 2.56 (0.869) | 2.84 (0.708) | 2.94 (0.753) | 2.96 (0.548) |  | 2.47 (0.680) |
| Geometric Mean (GCV%) | 2.38 (39.1%) | 2.51 (22.4%) | 2.64 (25.6%) | 2.41 (36.8%) | 2.75 (27.4%) | 2.84 (26.9%) | 2.91 (18.4%) |  | 2.37 (29.1%) |
| Median [95%] | 2.60  [0.966, 4.19] | 2.60  [1.76, 3.57] | 2.77  [1.65, 3.88] | 2.47  [1.24, 4.16] | 2.96  [1.67, 3.94] | 2.99  [1.81, 4.29] | 3.00  [2.10, 4.02] | 4.00  [4.00, 4.00] | 2.40  [1.30, 4.00] |

**Table S3. Endogenous BH_4_ Measurements in Patients with PKU**

|  | ≤2 years (N=10) | >2 years (N=39) | Overall (N=49) | P-value  (ANOVA) |
| --- | --- | --- | --- | --- |
| Mean (SD) | 4.49 (2.62) | 11.0 (5.27) | 9.68 (5.51) | 0.0005 |
| Geometric Mean (GCV%) | 3.80 (70.2%) | 9.63 (60.7%) | 7.96 (77.2%) |  |
| Median [95%] | 3.90 [1.40, 9.02] | 11.4 [2.98, 20.5] | 8.31 [2.15, 20.4] |  |

**
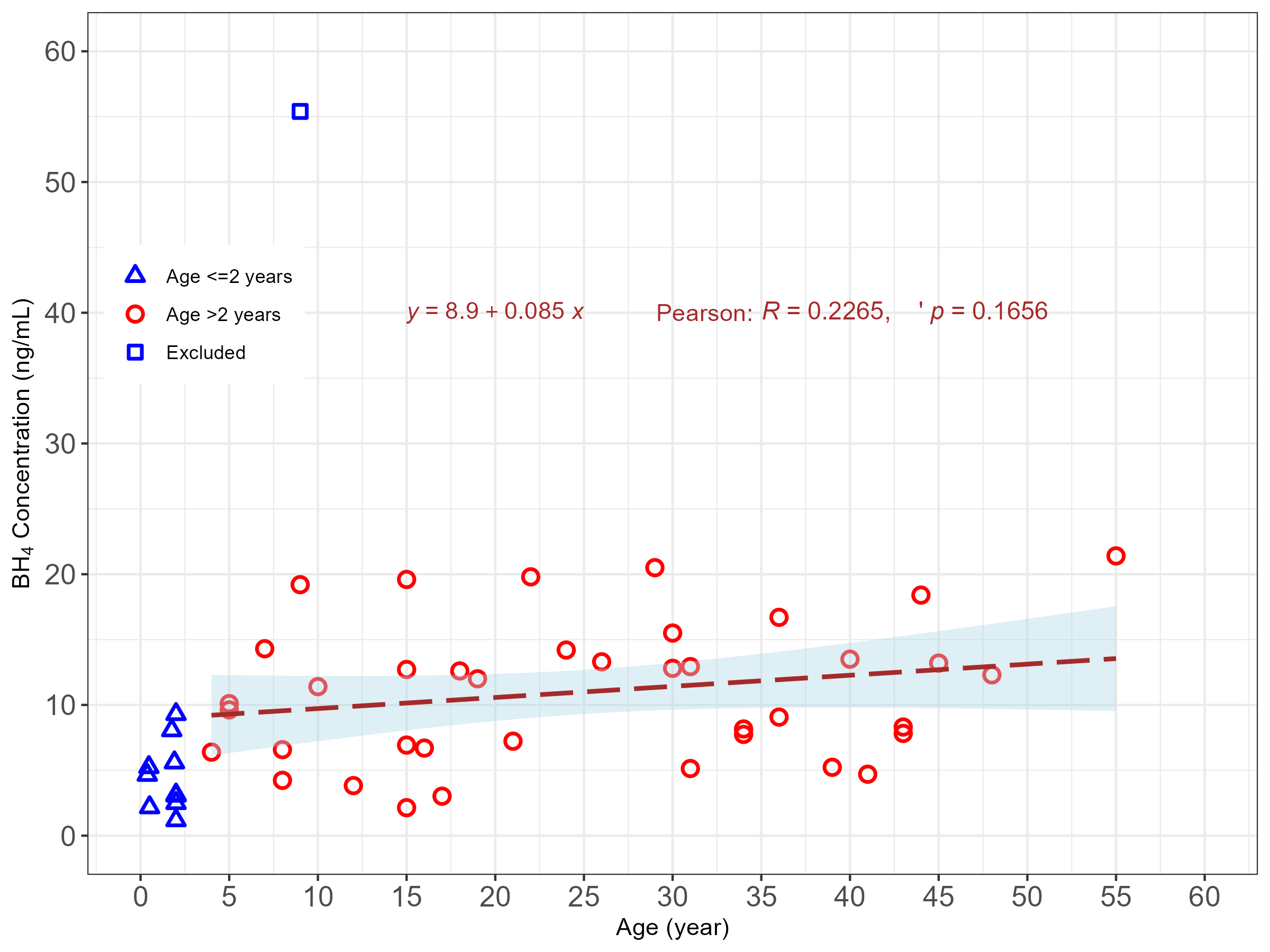
Figure S1. Endogenous BH_4_ versus Age in Patients with PKU**

**Figure S2. Endogenous BH_4_ Concentration in Patients with PKU Aged Above 2 Years**

**
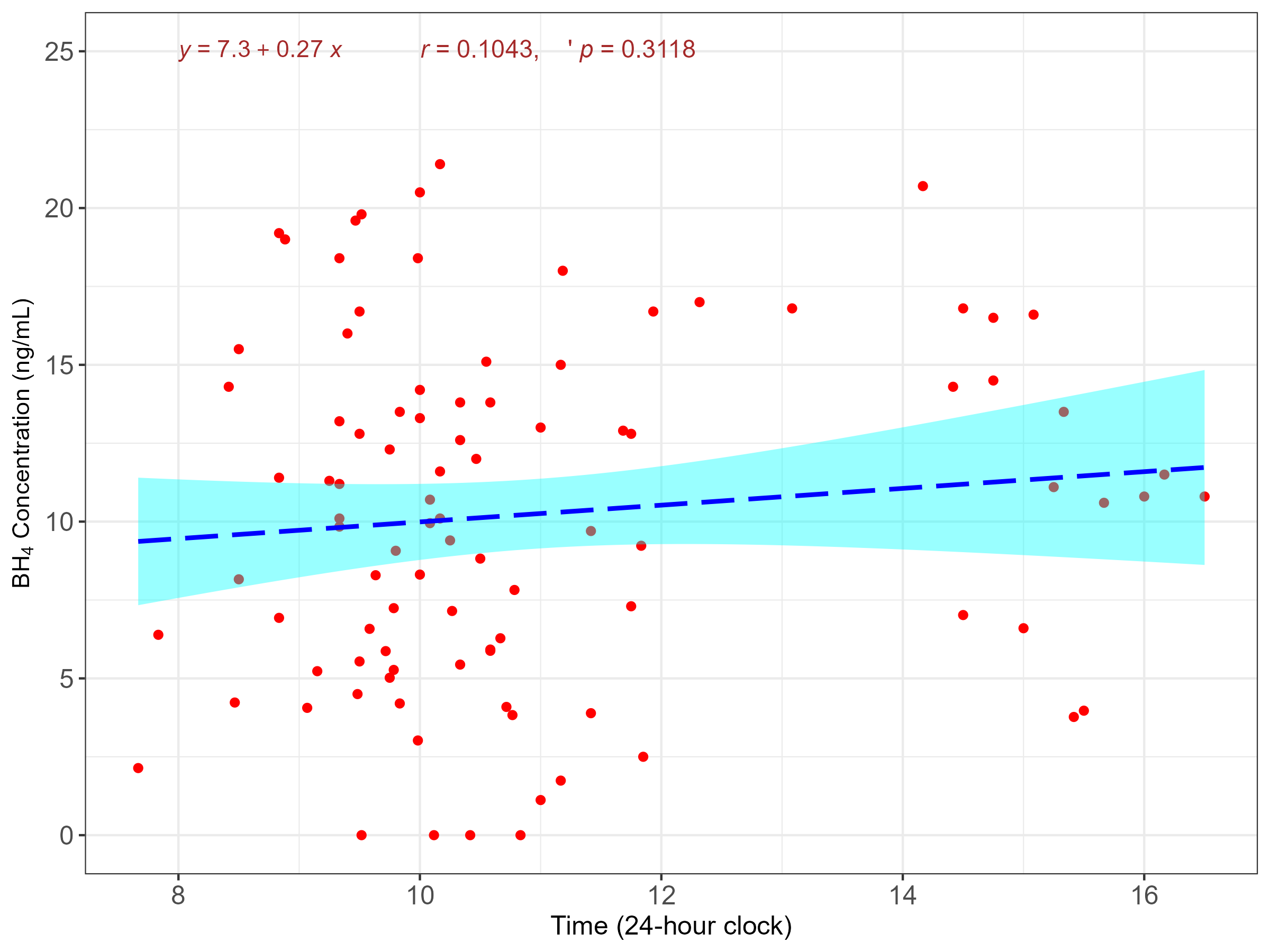
**
